# Analysis of an ankyrin-like region in Epstein Barr Virus encoded (EBV) BZLF-1 (ZEBRA) protein: implications for interactions with NF-κB and p53

**DOI:** 10.1186/1743-422X-8-422

**Published:** 2011-09-05

**Authors:** David H Dreyfus, Yang Liu, Lucy Y Ghoda, Joseph T Chang

**Affiliations:** 1 Department of Pediatrics, Yale SOM, 488 Norton Parkway, New Haven CT 06511, USA; 2 Department of Statistics, Yale University, P.O. Box 208290, New Haven CT 06520-8290, USA; 3 Keren Pharmaceutical, 63 Bovet Rd #143, San Mateo, CA 94402-3104, USA

**Keywords:** p53, NF-κB, transcription, ankyrin, phylogeny, oncogenes, viral carcinogenesis, viral conditioning, epi-genomics, ping-pong evolution, ASPP2, ASPP1, iASPP

## Abstract

**Background:**

The carboxyl terminal of Epstein-Barr virus (EBV) ZEBRA protein (also termed BZLF-1 encoded replication protein Zta or ZEBRA) binds to both NF-κB and p53. The authors have previously suggested that this interaction results from an ankyrin-like region of the ZEBRA protein since ankyrin proteins such as IκB interact with NF-κB and p53 proteins. These interactions may play a role in immunopathology and viral carcinogenesis in B lymphocytes as well as other cell types transiently infected by EBV such as T lymphocytes, macrophages and epithelial cells.

**Methods:**

Randomization of the ZEBRA terminal amino acid sequence followed by statistical analysis suggest that the ZEBRA carboxyl terminus is most closely related to ankyrins of the invertebrate cactus IκB-like protein. This observation is consistent with an ancient origin of ZEBRA resulting from a recombination event between an ankyrin regulatory protein and a fos/jun DNA binding factor. *In silico *modeling of the partially solved ZEBRA carboxyl terminus structure using PyMOL software demonstrate that the carboxyl terminus region of ZEBRA can form a polymorphic structure termed ZANK (ZEBRA ANKyrin-like region) similar to two adjacent IκB ankyrin domains.

**Conclusions:**

Viral capture of an ankyrin-like domain provides a mechanism for ZEBRA binding to proteins in the NF-κB and p53 transcription factor families, and also provides support for a process termed "Ping-Pong Evolution" in which DNA viruses such as EBV are formed by exchange of information with the host genome. An amino acid polymorphism in the ZANK region is identified in ZEBRA from tumor cell lines including Akata that could alter binding of Akata ZEBRA to the p53 tumor suppressor and other ankyrin binding protein, and a novel model of antagonistic binding interactions between ZANK and the DNA binding regions of ZEBRA is suggested that may be explored in further biochemical and molecular biological models of viral replication.

## Background

Analysis of viruses, mobile DNA sequences and host genomes reveals evidence that genetic information can be shared resulting in generation of novel hybrid genetic elements. For example, the ubiquitous human pathogen Epstein Barr Virus (EBV), genome structure contains a region co-linear with the vertebrate variable immunoglobulin gene segments including regulatory regions for the myc oncogene, signals for immunoglobulin switch recombination, somatic mutation and coding regions[1,2]. Similarly, the EBV viral termini contain sequences similar to somatic V(D)J immunoglobulin and T cell receptor recombination sites and the virus encodes a recombinase co-regulated with the host RAG V(D)J recombinase [3-5].

These observations suggest that EBV and other DNA viruses evolve through sharing of genetic information with the host genome termed "Ping Pong Evolution" [1,2]. In this work, a remarkable example of Ping Pong Evolution between EBV and the host immune system is characterized. The EBV viral protein ZEBRA, Zta encoded by BZLF-1is proposed to result from capture and fusion of exons from two different host immune response genes, the fos/jun transcription factor and IκB immune regulatory proteins. Analysis of host IκB proteins is utilized to present an empirically testable model of ZEBRA binding to NF-κB immune response proteins in which the unstructured carboxyl region of ZEBRA can assume two different structural conformations when bound to NF-κB. In addition, an amino acid polymorphism in this region of ZEBRA is identified that could potentially alter the functional properties of the ZEBRA protein interactions with both NF-κB and the distantly related tumor suppressor p53[6].

Studies of ZEBRA, the lytic switch protein, have demonstrated that the two amino terminal exons of ZEBRA are structurally related to the fos/jun transcription factors and that ZEBRA binding to DNA sites, known as ZRE, is sufficient to activate the viral lytic cycle[7,8]. The carboxyl region of ZEBRA protein can bind to components of the NF-κB transcription family *in vitro *and alter NF-κB transcription *in vivo*[9,10]. Inactivation of NF-κB transcription is apparently independent of ZEBRA's ability to function as a transcription factor [11-13]. It is not currently known which of these multiple effects of ZEBRA contribute to viral carcinogenesis in a humanized mouse model [14].

One potential outcome of these interactions is that inactivation of NF-κB transcription during viral lytic replication confers a selective advantage upon the virus [15,16]. NF-κB transcription factors and regulatory IκB proteins are central mediators of both the innate and acquired immune responses [17,18]. Inactivation of NF-κB could block the innate immune response in B lymphocytes which are the viral host cell. Interaction between ZEBRA and NF-κB can trigger apoptosis of cells expressing ZEBRA [19-21]. Inactivation NF-κB transcription by ZEBRA in host B lymphocytes may also oppose the effects of latency proteins that activate NF-κB transcription, delaying apoptosis and contributing to viral maturation and release from pre-apoptotic host cells.

Other bystander cells such as T-lymphocytes, epithelial cells and macrophages may also be transiently infected with EBV. T lymphocytes in particular express both the EBV CD21 receptor as well as other EBV receptors and express ZEBRA protein [3,21,22]. Studies of the effects of transient and stable expression of ZEBRA in T-lymphoblastoid cell lines have confirmed an IκB-like inactivation NF-κB signaling by ZEBRA and increased T lymphocyte apoptosis [21]. Thus, an additional selective advantage of interactions between ZEBRA and NF-κB may be destruction of immune responder cells through transient infection and apoptosis.

Another human gamma herpesvirus, human herpesvirus-8, first identified as the cause of Kaposi's sarcoma, encodes a lytic replication protein which lacks DNA binding but has extensive amino acid similarity to ZEBRA including the carboxyl region of ZEBRA [23-25]. This region of ZEBRA is highly conserved between different viral strains [26]. EBV infected lymphocytes also express a trans-spliced ZEBRA like protein termed RAZ sharing the carboxyl terminal NF-κB binding region of the ZEBRA protein, but lacking the ability to bind DNA or ZRE[15,27]. RAZ is also expressed in EBV infected T lymphocytes[3,22].

Remarkably, the carboxyl region of ZEBRA required for interactions with NF-κB transcription and ZEBRA dimerization also binds to p53 tumor suppressor *in vitro *and alters p53 transcription *in vitro*[28-30]. The effects of ZEBRA on p53 transcription are stimulatory in some cases such as T-lymphocytes and epithelial cells, but inhibitory in B-lymphocytes[29,30]. Interactions between ZEBRA and p53 also involve other p53 binding proteins and are more complex than interactions *in vivo *between ZEBRA and NF-κB [31]. The ability of ZEBRA to interact with both NF-κB and p53 has previously been suggested to result from a cryptic IκB-like region in the carboxyl terminus of the ZEBRA protein[6,21]. NF-κB and p53 proteins share a common IκB binding region because they are descendents of a common ancestral transcription factor previously termed "proto p53/NF-κB[6]". Since this hypothesis was proposed, the crystal structure of ZEBRA protein has been partially solved including a portion of the carboxyl terminus of the protein interacting with NF-κB and p53[8,32]. In addition, the interactions between p53 binding ankyrin proteins and NF-κB proteins have been characterized providing additional evidence that regulatory proteins in the ankyrin family, including NF-κB inhibitor proteins related to IκB, bind to both NF-κB and p53 proteins[33].

The recently available partial structure of the ZEBRA carboxyl terminus, and the structures of IκBα and apoptosis-stimulating protein of p53 (ASPP2, previously known as p53BP2) ankyrin proteins are analyzed in this work, building on previous similarities noted between these regulatory proteins[6]. First, it is demonstrated that primary amino acid similarities between the alpha helix regions of ZEBRA and various other ankyrin proteins are unlikely to have arisen by chance or independent parallel evolution but instead appear to represent capture and homologous descent of a terminal exon encoding an IκB domain by ZEBRA. Second, it is shown that the partial crystal structure of ZEBRA is consistent with dimerization of the terminal region of the protein to form an IκB-like stem and loop structure. Finally, individual ankyrin domains of IκBα are shown to have structural similarities to the ankyrins of ASPP2, and thus able to bind to the same region targeted by ZEBRA[6].

## Methods

### Definition of the ankyrin-like region of ZEBRA protein termed "ZANK"

**Figure 1:**
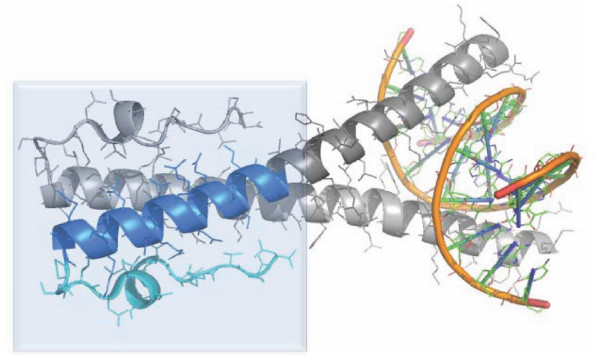
**Structure of ZEBRA protein**. The carboxyl terminus of dimeric ZEBRA protein bound to a ZRE oligonucleotide (orange) is shown. This image was generated from previously determined ZEBRA molecular coordinates using PyMOL. The ZANK (ZEBRA ANKyrin-like region) carboxyl terminus of ZEBRA is highlighted in blue. ZANK is composed of two regions, a highly structured alpha helix stem highlighted in darker blue (the ZANK stem region) and a partially solved unstructured region continuing to the end of the protein highlighted in light blue (the ZANK loop region).

**Figure 2:**
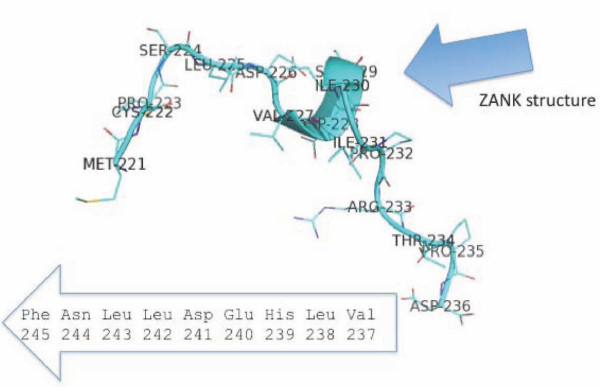
**Sequence and Structure of ZANK**. The amino acid sequence of the unstructured loop region of ZANK (shown in light blue in Figure 1) is shown in more detail including the addition of terminal amino acids 237-245 not present in the solved structure. This region was deleted in order to permit crystallization of the ZEBRA protein. A region with secondary structure denoted "ZANK structure" is evident in the carboxyl terminus of ZEBRA as discussed in more detail in the text. ZANK is encoded by a discrete exon corresponding precisely with the stem and loop regions shown in blue in Figure 1.

**Figure 3:**
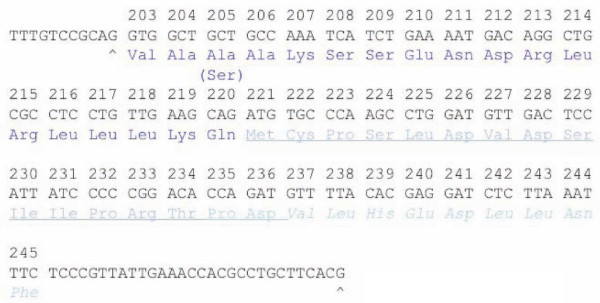
**ZANK region amino acids correspond precisely with exon 3 of BZLF-1 gene encoding ZEBRA**. DNA encoding the ZANK region and mRNA splice sites are shown. Using the same color scheme as in Figure 1, amino acids corresponding to the ZANK stem are shown in dark blue and those corresponding to the loop region are shown in light blue. DNA sequence is derived from the B-958 strain of EBV. A single amino acid polymorphism at aa 205 in this region has been identified resulting in a Ser substitution in certain strains of EBV such as Akata. Underlined sequences indicate regions of the putative ZANK loop present in the crystal structure. The splice points of ZEBRA exon 3 encoding ZANK is shown as "^" starting at the ZEBRA amino acid sequence "Val-Ala-Ala-Ala..." continuing to the end of the protein. The structured region of ZANK loop (Figure 1, 2) corresponds to ZANK sequences 227-230 Val-Asp-Ser-Ile. Residues His239 and Phe245 in the terminus of ZANK correspond to functionally conserved residues in the loops of other ankyrin proteins as discussed in more detail in the text.

**Figure 4:**
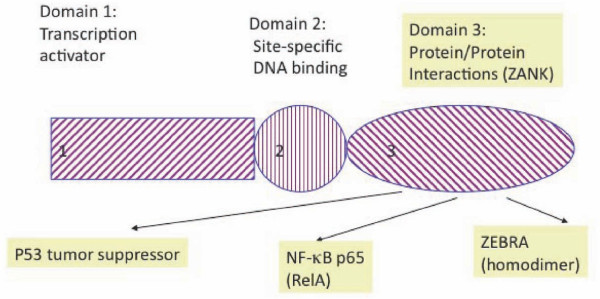
**A schematic diagram of the ZEBRA protein illustrating the 3 functional domains of ZEBRA**. ZEBRA domains 1 and 2 contain the transcriptional activation and DNA binding regions of the protein, respectively, and are similar to Fos/Jun transcription factors. Domain 3, the carboxyl region of ZEBRA denoted ZANK in this work, is required for both dimerization of the ZEBRA protein as well as interactions with NF-κB and p53 transcription factors.

We previously noted that a region of ZEBRA (Figures 1, 2) has primary amino sequence similarity to IκB prior to the determination of the crystal structure of ZEBRA [6]. We first used the program PyMOL (Delano Scientific Inc., CA) to define and visualize a region of ZEBRA whose primary amino acid sequence is similar to ankyrin proteins [6]. ZEBRA structure was partially solved by x-ray crystallography in 2006 and structural coordinates are available as a protein data-base in the public domain (2C9N.pdb). This region of ZEBRA, termed in this work ZANK "ZEBRA ANKryn-like region" has an alpha-helical stem and carboxyl unstructured region (Figure 1). In the course of this analysis, The ZANK region of ZEBRA was noted to be encoded by a single exon in all examined EBV strains coinciding exactly with the exon-intron junction of the ZEBRA protein third exon (Figure 2, 3). Thus, the p53 and NF-κB binding regions of ZEBRA, termed ZANK, were found to be separate both structurally and genetically from the DNA binding and trans-activation regions of ZEBRA (Figure 4).

### Alignment of ZANK with defined ankyrin proteins

**Figure 5:**
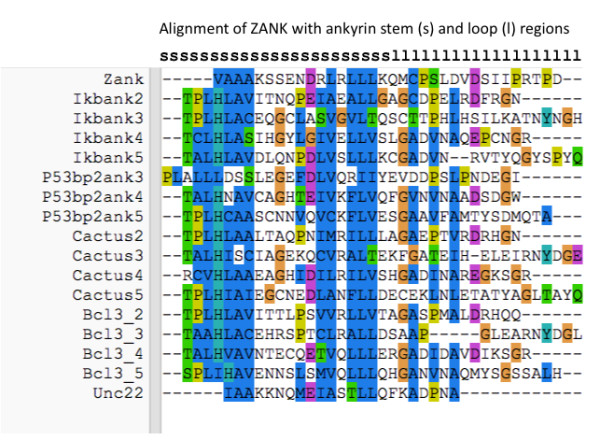
**Alignment of ZANK amino acid sequence with vertebrate and invertebrate anykrins**. ZANK amino acids present in the solved ZEBRA structure are shown aligned with corresponding regions of non-viral ankyrins. Alignments were performed using clustalx, and individual vertebrate ankyrin stem loop domains from IκB, p53BP2/ASPP2, BCL3, and invertebrate ankyrins Unc-22 and Cactus. Approximate borders of stems (s) and loops (l) derived from PyMOL generated structures are shown at the top of the figure. Colored regions indicate highly conserved or identical amino acids according to the color scheme described at http://ekhidna.biocenter.helsinki.fi/pfam2/clustal_colours.

ZEBRA protein and nucleotide sequences are as determined previously[26]. Individual ankyrin domains from published IκB and related sequences obtained from genbank were identified using PyMOL and first aligned with ZANK and with each other using standard algorithms such as BLAST as illustrated in the text (Figure 5) Color scheme for Figure 5 can be found at http://ekhidna.biocenter.helsinki.fi/pfam2/clustal_colours). Previously, some primary amino acid similarity between p53 binding ankyrins, IκB ankyrins, invertebrate ankyrins and the ZEBRA carboxyl terminus have been illustrated and suggested to provide a basis for interactions between ZEBRA and ankyrin binding proteins[6,21]. Individual ankyrin domains in ASPP2 are denoted in this work based on previously reported optimized alignments between the p53BP2 fragment of ASPP2 and IκB. Thus, ank3 of p53BP2 corresponds to IκB ankyrin 3, p53BP2 ank4 corresponds to IκB ankyrin 4, and p53BP2 ank5 corresponds to IκB ankyrin 5 proceeding from the amino to carboxyl termini of both proteins respectively[6]. In a more recent naming convention, p53BP2 which was found to be a fragment of a larger protein known as ASPP2 (ankyrin repeat, SH3, and proline rich domain-containing protein number 2 also previously called apoptosis stimulating p53 binding protein 2) to indicate that it is a member of a multi-protein family. Using this nomenclature, p53BP2 ank3 corresponds to ASPP2 ankyrin 1, p53BP2 ank4 corresponds to ASPP2 ankyrin 2, and p53BP2 ank5 corresponds to ASPP2 ankyrin 3 [34].

### Randomization analysis of ZANK and other defined ankyrin domains

**Figure 6:**
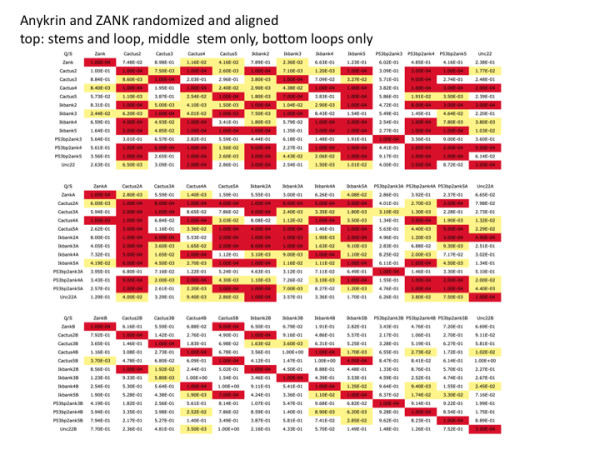
**Statistical analysis of ZANK amino acid sequence similarity to other ankyrins**. Representative results of shuffling experiments for ZANK, vertebrate ankyrins, IκBα, p53BP2/ASPP2 ankyrins, and invertebrate ankyrins Cactus and Unc-22 are shown. Top: Randomizations of whole ankyrin repeats. Middle: ankyrin stem regions only. Bottom: ankyrin loop regions only. 10,000 randomly shuffled sequences of identical amino acid composition were generated in each cell. Shuffled sequences were aligned with indicated non-shuffled sequence and the number of scores greater than or equal to that of the non-shuffled input sequence is shown.. Significant alignments (p < 0.05) are shown highlighted in yellow while highly significant alignments (p < 0.001) are shown highlighted in red.

A randomization shuffling strategy was developed to estimate the probability that observed amino acid similarities between ZANK, IκB and other related ankyrins such as invertebrate cactus and unc-22 were not explained by the non-random amino acid composition of ankyrin proteins. The amino acid sequence of each ankyrin was randomized or shuffled and compared to every other possible sequence with the number of equal or better scores to non shuffled sequence shown. Repeated IκB and p53BP2 units of ankyrin are composed of a rigid alpha-helical stem and a less structured more variable loop region that may be under different evolutionary constraints. The borders of each ankyrin stem and loop were identified using PyMOL (Figure 5). Because of this bi-partite structure, analysis of the stem and loop regions was conducted both together and independently (Figure 6). Randomization of 10,000 of each stem and loop region was used in this analysis with correction for multiple comparisons. In this analysis, alignment of less than 500/10,000 randomized sequences is comparable to a P value of .05, shown in yellow. Alignment of less than 10/10,000 sequences comparable to a P value of .001 is shown in red.

Based on these studies the similarity between putative ZEBRA ankyrin stem and the cactus ankyrin stems 4 is one order of magnitude greater than other ankyrin stems, and this relationship was maintained over a variety of Price Waterman gap values. Also highly unlikely to occur by chance are the alignments between the putative ZEBRA ankyrin stem and the cactus stem 2 and Bcl3 ankyrin 4 stem. In many cases individual ankyrin stems from different ankyrin proteins such as invertebrate cactus, IκB and p53 binding protein were more closely related than adjacent ankyrins from a single protein, for example cactus ankyrin stem 4 and bcl3 ankyrin stem 4 are more similar than cactus ankyrin stem 4 and other cactus ankyrins (BCL3 data not shown). Based upon randomized sequence comparisons shown in the ZEBRA stem-like region is most similar to the stem region 4 of a Cactus ankyrin from the *Drosophila *fruit fly. Interestingly, the loop region of ZEBRA is most similar to a different loop region from the cactus protein, ankyrin 5 of cactus. However, because of the short length o the ZEBRA stem region and other confounding factors such as apparent functional constraints on stem sequence evolution it was not possible to construct a unique rooted phylogeny of individual ankyrin stems using currently available alignment software (data not shown).

Visual inspection of individual ankyrin stem and loop regions (Figure 5) also suggests that the loop regions of all of the cellular ankyrins were less significantly related either to ZEBRA or to each other except in the stem regions. In addition, IκB and cactus ankyrins follow a pattern where alternating ankyrin loops are similar between odd and even numbered repeats, for example ankyrin 3 and 5 loops have more similarity in both amino acid composition and length than to ankyrins 2 and 4 loops from both IκB and cactus. Relationships between individual ankyrin stems and loops thus appear to represent a complicated mixture of functional constraints operating independently of evolutionary descent and shared sequences conserved through descent from a common precursor as will be analyzed in more detail elsewhere.

### In silico modeling of structural similarities between ZANK and other defined ankyrin domains

Protein data based files of ZEBRA (2C9N.pdb), IκB bound to NF-κB protein p65 (1lKN.pdb), and the p53BP2 fragment of ASPP2 (1YCS.pdb) were used to generate PyMOL based alignments. Experimentally determined structures were aligned using the "align" feature of PyMOL without any operator adjustments and using molecular coordinates as deposited in respective pdb files without alteration of molecular coordinates. ZANK was superimposed upon adjacent ankyrin domains of the IκBα protein initially solved as a complex with NF-κB p65 and the p53BP2 fragment of ASPP2 p53 binding protein solved as a complex with p53 (p53 structure not shown). A mechanism is proposed consistent with the partial structure of ZANK suggesting that the observed structure of ZANK could act as a dimorphic dimer and interact with adjacent dimorphic regions of NF-κB, and also related ankyrin binding regions of p53. IκBα and IκBα and p53 binding ankyrin proteins, although not identical in primary amino acid sequence, have remarkably similar interactions with NF-κB protein. This analysis is also used to suggest a testable hypothesis where an amino acid polymorphism previously identified between ZEBRA protein from Akata tumor cells and the ZEBRA protein from non-tumorigenic B-958 could modulate the binding interactions between ZEBRA and p53 leading to a more cancer-promoting viral phenotype in the case of the Akata derived ZEBRA.

## Results

### Identification of a structural and functional divergence between ZEBRA carboxyl terminus and fos/jun transcription factors

Epstein Barr Virus (EBV) encoded ZEBRA protein is capable of switching the viral state from latency to lytic growth through its effects on specific DNA binding sites termed ZRE (ZEBRA Response Elements)[7]. The crystal structure of the protein bound to a ZRE oligonucleotide reveals similarity to Fos/Jun and other DNA binding proteins of the BZIP transcription family as expected from similarities between ZRE and AP-1 binding sites[8]. The structure of the carboxyl terminal region of the ZEBRA protein has not been determined in the published crystal structure as inclusion of this region prevented protein crystallization suggesting a disordered structure (Figure 1) [32]. This region of ZEBRA is also dispensable for DNA binding[11].

Efforts to discover a specific mechanism for protein-protein interactions of the carboxyl terminus region of ZEBRA based upon the partially solved structure have been unsuccessful, although some similarities are evident between this region and C/EBP-α, a CAAT-binding transcription factor whose dysregulation is implicated in acute myeloid leukemia [13]. Notably, the conserved carboxyl terminus of ZEBRA (Figure 2) diverges from DNA binding proteins of the BZIP family precisely at the intron border of exon 3 (Figure 3). Like the ZEBRA terminal exon, IκB binds to both p53 and NF-κB [6,21]. Exon 1 and 2 of ZEBRA thus encode a Fos/Jun homologue that activates DNA transcription to initiate the viral lytic program, while exon 3 (shared with trans-spliced RAZ protein) provides both protein self-dimerization and binding to other master regulatory transcription factors such as p53 and NF-κB. These observations are analyzed in more detail in the remainder of this work.

### ZANK, the carboxyl terminal Zebra ANKyrin-like region

Interactions between ZEBRA and NF-κB, p53 as well as other proteins can occur independently of ZEBRA DNA binding, since experiments *in vivo *have shown that in T-lymphocytes ZEBRA is not bound to DNA but localized to the cytoplasm where it blocks NF-κB translocation to the nucleus [6,21]. The full sequence of the carboxyl terminus exon (exon 3) of ZEBRA is shown in Figure 2, including both regions of the protein present in the crystal structure (blue highlights in Figure 1) and extreme carboxyl residues not present in the crystal structure [26]. We previously suggested that the carboxyl terminus of ZEBRA encodes an ankyrin-like structure responsible for the ability of ZEBRA to bind ankyrin binding proteins NF-κB and p53 [6,21]. For purposes of discussion, the carboxyl terminus of ZEBRA encoded by exon 3 is termed ZANK (for ZEBRA ANKyrin-like region) in the remainder of this work. Remarkably, the crystal structure of ZEBRA protein (Figure 1) shows divergence from BZIP Fos/Jun DNA binding proteins precisely at the intron/exon borders of ZEBRA exon 3 (Figure 2, 3).

### Comparison of ZANK with defined vertebrate and invertebrate ankyrin domains

ZANK spans part of the alpha helix of ZEBRA and unstructured regions at the carboxyl terminus of the protein (Figure 3, 4). The alpha-helical stem-like regions contribute most of the similarity between ZANK and individual ankyrins (Figure 5). This is evident in Figure 5 because most of the colored coded similar regions occur to the left of the figure in the stem regions (denoted s) rather than the loop regions (denoted l). This was confirmed by a randomization analysis (Figure 6). Randomizations demonstrate that the full ZANK sequence or loop region results in less significant alignments or no significant alignments for both ZANK and other ankyrins compared to randomization of the stem regions (Figure 6). The alpha helical regions of ankyrin binding proteins are highly structured to provide a rigid framework, while the loop regions are less structured allowing for binding to different substrates. Results are consistent with the hypothesis that ZANK encodes amino acids derived from a common ancestral ankyrin domain shared with IκB and p53 binding ankyrins and are most closely related to the invertebrate *Drosophila *ankyrin regulatory protein Cactus, rather than vertebrate ankyrins.

Statistical significance as highlighted in yellow in Figure 6 is defined as a given alignment found in less than 5% of shuffled sequences (less than 500 out of 10,000), corresponding to P < 0.05 that the alignment would occur by chance. A more stringent definition of a non-random alignment (highlighted in red in Figure 6) is defined as a given alignment found in less than 0.1% of shuffled sequences (less than 10 out of 10,000). In all cases analyzed, including ZANK, the stem or alpha helix regions of ankyrins are more conserved across ankyrin protein families than the loop regions. For example, not all IκBα loops in host proteins are similar to each other, but all the IκBα stem regions are similar based upon 10,000 random shuffling of the respective sequences.

The diagonal of each randomization experiment shown is the identity of the sequence with itself shown in red, which as expected, occurs once per 10,000 shuffling experiments. Because each cell represents a characterization of an independent shuffling "experiment" the number of aligned sequences are slightly different even for identical comparisons as can be seen by comparing identical comparisons on either side of the diagonal identity but because of the large number of shuffling per "experiment" results are usually similar for a given cell on either side of the diagonal.

### ZANK is more similar to an invertebrate ankyrin domain from Cactus than vertebrate ankyrins

As shown in Figure 6, the ZANK stem was most similar to the ankyrin 4 stem region of invertebrate *Drosophila *Cactus regulatory protein at the highest level of significance (less than 10/10,000 of shuffled sequences). ZANK loop was most similar to invertebrate *Drosophila *Cactus regulatory protein ankyrin 5 loop region at the highest level of significance (less than 10/10,000 of shuffled sequences). Similarity was also evident between ZANK and the Cactus regulatory protein ankyrin 2 stem region and the IκBα ankyrin 3 and 5 stems as well as the whole protein. Similarity to both stem and loop regions of Cactus in addition to elements of IκBα is a surprising result since EBV is a vertebrate pathogen, while Cactus is an invertebrate host gene, suggesting that the divergence of ZANK from other ankyrins was ancient, possibly preceding divergence of p53 and NF-κB ankyrin binding proteins such as IκBα. Alternatively, a vertebrate virus encoding ZANK could have resulted from recombination with an invertebrate *Drosophila *virus since in some cases insect viruses can infect vertebrate cells. Existing phylogeny programs do not appear able to resolve these two possibilities as will be discussed in more detail elsewhere.

### *In silico *Modeling of ZANK

In the remainder of this work we used PyMOL generated structures to model experimentally observed binding between ZEBRA protein and both NF-κB and p53 *in vitro *and *in vivo*. A limitation of this analysis is that the existing structure of ZANK is both partial and also solved in the absence of the partner ligands p53 or NF-κB. However, even with those limitations this analysis is sufficient to suggest a model of ZANK interactions with its partner ligands in which the ZANK loop adopts two different conformations. We have previously suggested that binding between ZEBRA carboxyl terminus and other proteins can be explained by the common descent of both NF-κB and p53 from an ancestral protein termed proto-p53/NF-κB. If this hypothesis is correct then it should be possible to identify a common structural signature or code between ZANK, NF-κB binding ankyrins and p53 binding ankyrins that ZANK has evolved to fit into. To use an analogy, if ankyrins resemble a key that can bind to their substrates resembling a lock, then the ZANK key should fit into both the NF-κB and p53 locks[6,21].

**Figure 7:**
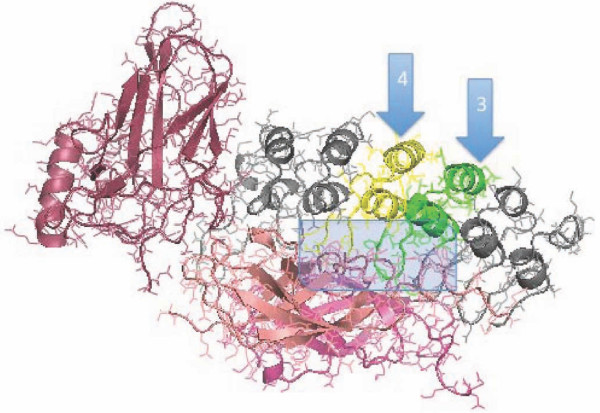
**Illustration of the Interaction of IκBα ankyrins with NF-κB**. NF-κB p65/p50 dimer subunits (red shadings) are shown bound to ankyrin repressor IκBα. IκBα ankyrin domains 3 (green) and 4 (yellow) are shown in color while other regions of the IκBα protein are shown in grey. Each ankyrin unit is composed of a structurally conserved alpha helix dimer backbone, and a less structurally conserved loop region inserting into the NF-κB p65/p50 groove.

To explore the possibility of this "lock and key" mechanism, IκBα ankyrin structures were extracted and superimposed as shown, without any other alterations in the solved crystal structure coordinates. Since ZEBRA protein exists as a dimer of identical subunits (Figure 1), two adjacent ZANK loop domains in the dimerized ZEBRA protein could interact with adjacent IκBα ankyrin binding regions of NF-κB. As shown, Ank 3 and 4 of IκBα play a critical role in IκBα interactions with NF-κB (Figure 7). In support of this hypothesis, residues such as Histamine 239 in ZANK (Figure 3), not present in the crystal structure of ZEBRA due to instability in the absence of either co-ligand NF-κB and tumor suppressor p53, could align with a corresponding conserved stem histidine in IκBα Ank 3 and related ankyrin proteins (Figure 5). Binding to proteins such as NF-κB and p53 could stabilize ZANK and permit a crystal or structure to confirm this model.

### NF-κB binding ankyrins have an alternating key or code of short and long ankyrin loops suggesting a polymorphic ZANK loop domain

**Figure 8:**
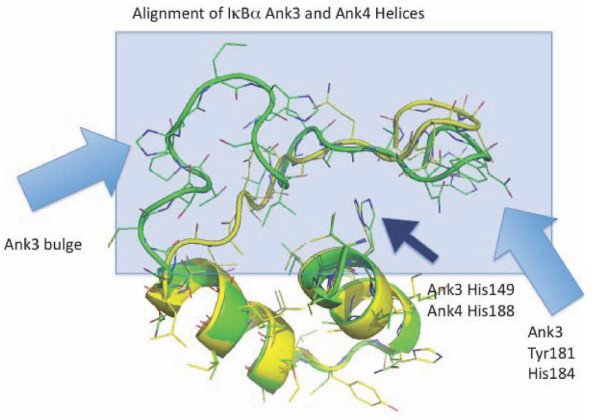
**Alignment of IκBα Ank3 and Ank4 Helices**. IκBα Ank3 (green) and Ank4 (yellow) domains are extracted from the NF-κB p65/p50 dimer bound structure and are shown superimposed to illustrate the highly conserved structure of the stem and the less conserved loop region. Ank3 loop has a bulge not present in Ank4. Ank3 also has more bulky hydrophobic residues than Ank4 in the region of the loop extending most deeply into the NF-κB p65/p50 structure. Arrows illustrate key features of Ank3 and Ank4 including a highly conserved His residue shared by most ankyrin stems (Ank3, His 149; Ank4, His 188). Also shown are bulky residues in Ank3 (Tyr 181 and His184) present in Ank4.

**Figure 9:**
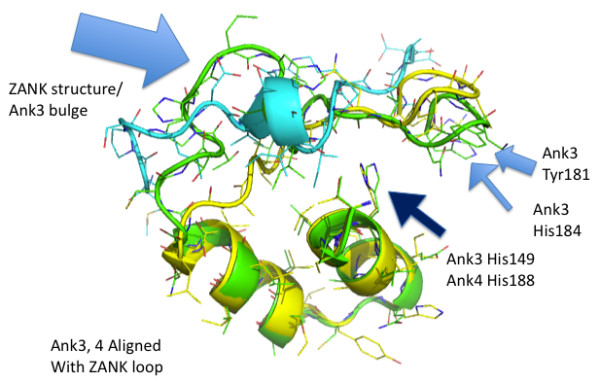
**Alignment of ZANK Loop with IκBα Ank3 and Ank4**. The structure of ZANK loop (light blue) is superimposed on IκBα Ank3 (green) and Ank4 (yellow) structures. The structured region present in ZANK loop overlaps the bulge of Ank3, not present in Ank4. Like Ank4, ZANK has no significant hydrophobic regions in the solved structure, but ZANK extends further due to unstructured residues not present in the solved structure (Figure 2).

**Figure 10:**
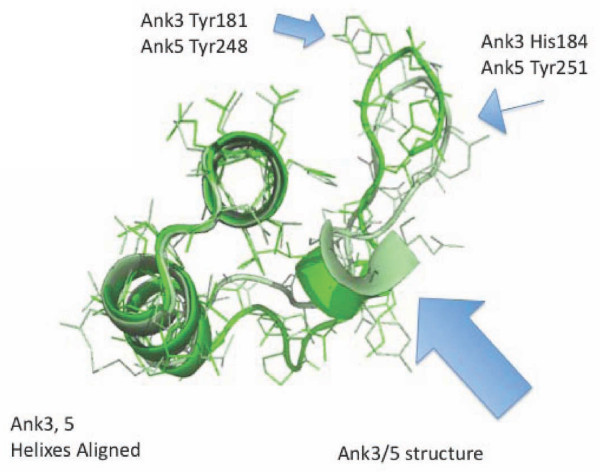
**Structurally similar large ankyrin loops alternate with small ankyrin loops in IκBα**. IκBα Ank3 (green) is shown superimposed on IκBα Ank5 (light green) to illustrate corresponding structural features. Alternating small and large ankyrin loops seem to be a conserved feature of vertebrate and invertebrate NF-κB binding ankyrins, suggesting a "lock and key" mechanism. ZEBRA is proposed to mimic both large ankyrin loop structures and small ankyrin loop structures in order to fit into the NF-κB binding code. The bulge region of Ank3 and Ank5, although encoded by different primary amino acids both can form a structure corresponding to the helix structure found in ZANK (Figure 9). IκBα Ank3, Ank5, and potentially ZANK, also share conserved orientation of aromatic and bulky amino acids in the loop regions not present in IκBα Ank4.

Ankyrin loop regions are variable in length while the stems are conserved in length (Figure 5). There is an alternating pattern of short loops (approximately 15 amino acids between adjacent stems) and long loops (approximately 20 amino acids between adjacent stems) in all of the NF-κB binding ankyrins including vertebrate IκBα and invertebrate cactus. Thus both IκBα and cactus ankyrin loops 2, 4, and 6 are short and loops 1, 3, and 5 are long. To permit binding interactions with NF-κB, the ZANK loop region of ZANK would need to be dimorphic or sufficiently unstructured or flexible to conform to adjacent non-identical short and long ankyrin binding pockets in NF-κB, for example the regions of NF-κB binding to Ankyrins 3 and 4 of IκBα as shown (Figure 7, 8, 9). Because the ankyrin loops of IκBα ank5 are very similar in both primary and secondary structure to IκBα ank3, IκBα has an alternating pattern of Ank3 and Ank4-like subunits, and this alternating structure could permit multiple ZANK binding sites in a single NF-kb ligand (Figure 10).

Also resulting from this alternating short loop/long loop pattern, using PyMOL generated structures it is evident that IκBα ankyrin 3 has additional amino acids in its loop region forming a bulge not present in ankyrin 4, and also has hydrophobic amino acids in its loop not present in ankyrin 4 (Figure 9). If ZEBRA were to bind to both IκBα ankyrin 3 and 4 binding regions in NF-κB, ZANK would need to adapt 2 different conformations to fit into the two different sites, for example by having free amino acids outside of the binding region at the Ankyrin 4 site, while utilizing additional amino acids in the extreme carboxyl region of the ZEBRA protein in the deeper Ankyrin 3 site. The bulge region present both ankyrin 3 and 5 loops is not present in ankyrin 4 loop (Figure 10), but corresponds to a structured region of the ZANK loop (Figure 9). In the IκBα structure, these structured regions of are proposed to form a α helix like region stabilizing the IκBα loops.

### Shared structural elements between p53 and NF-kB binding ankyrins identified as a potential binding mechanism of ZANK

**Figure 11:**
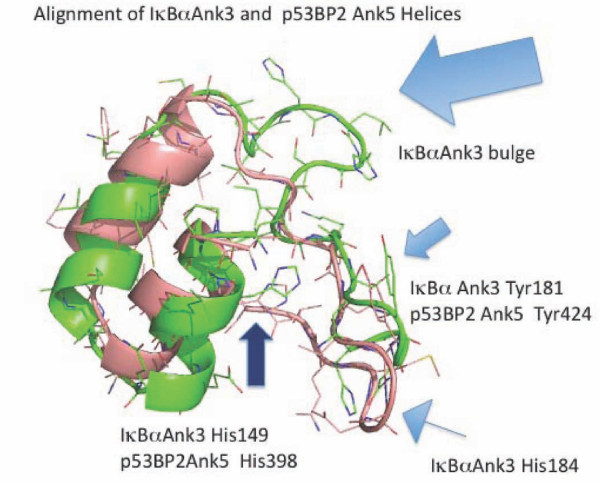
**Alignment of IκBα Ank3 and p53BP2/ASPP2 Ank5 structures are consistent with a shared binding mechanism between NF-κB binding ankyrins and p53 binding ankyrins that could be targeted by ZANK**. IκBα Ank3 (green) is shown superimposed on p53bp2/ASPP2 Ank5 (Salmon). P53bp2/ASPP2 Ank5 is similar in length to NF-κB binding short ankyrin loops but also shares structural features spatially conserved in location and orientation to long ankyrin loops such as IκBα Ank3. p53BP2/ASPP2 Ank5 aa Tyr 424 aligns structurally a with IκBα Ank3Tyr 181 (filled blue arrow). The terminal residue of ZANK (Tyr 245) could substitute for highly conserved aromatic tyrosine binding interaction between IκBα Ank3 tyr 181 and NF-κB, as well as coordinating corresponding interactions between ZANK and p53. Both short and long ankyrins and P53bp2/ASPP2 Ank5 share a highly conserved histidine corresponding to IκBα Ank3 aa His 149 (dark blue arrow). A corresponding residue is present in ZANK (Val 203). P53bp2/ASPP2 Ank5 lacks a residue corresponding to IκBα Ank3 aa His 184 (narrow stem blue arrow), but a corresponding residue (His 239) is present in ZANK.

We suggested previously that that the ability of ZEBRA to bind both NF-κB and p53 results from an evolutionarily conserved binding pocket shared between the two proteins[6,21]. The solved structure of p53bp2 (more recently denoted ASPP2 (1YCS.pdb)) bound to p53 demonstrates that p53bp2/ASPP2 contacts p53 in only the extreme carboxyl terminus, ankyrins 4 and 5, of its ankyrin regions, although other binding modes could exist *in vivo*. Pymol generated alignment between IκBα ankyrin 3 and p53bp2/ASPP2 ankyrin 5 is shown (Figure 11). p53bp2/ASPP2 ankyrin 5 has a structure with features similar to IκBα ankyrin 3 -- a highly conserved tyrosine is shared between ankyrin 3 and ASPP2 ankyrin 5, and this residue in IκBα is known to form a specific contact with a tyrosine in NF-κB p50/p65 dimer. Interestingly, the p53bp2/ASPP2 ankyrin loops do not have a short long loop pattern (Figure 5) but instead have a length corresponding to the short IκBα ankyrin loops (approximately 15 aa), and a conserved tyrosine typical of the long loops. These observations are consistent with a shared binding signature between some NF-κB and p53 binding proteins that could be targeted by a polymorphic ZANK loop as a master key.

### Identification of a cancer associated sequence variant in ZANK potentially altering p53binding interactions

**Figure 12:**
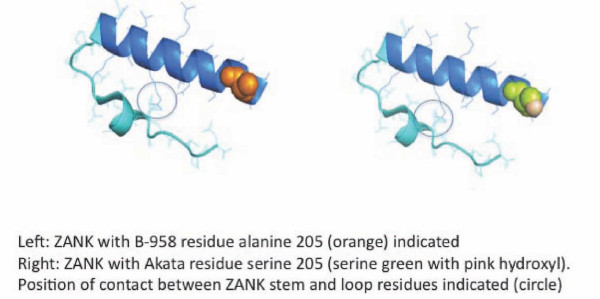
**ZANK Structure with S205A polymorphism illustrated**. A functionally conserved polymorphism in ZANK, A205 is altered to Ser is present in some EBV positive lymphoma cell lines such as Akata. A similar serine rather than alanine residue is present in some p53 binding protein ankyrin domains but not in NF-κB binding ankyrin domains. Changes in the stem or loop regions of ZANK could alter ZANK loop interactions with p53 and NF-κB particularly if covalently modified by serine phosphorylation. The circled region indicates a potential interaction between ZANK stem and loop amino acids.

An advantage of PyMOL generated *in silico *structural analysis of ankyrin domains in NF-κB proteins and p53 binding proteins is that it may now possible to predict the behavior of polymorphisms in ZEBRA that increase or decrease the relative strength of binding to NF-κB versus p53 proteins (Figure 12). Alterations in ZANK amino sequences could alter the ability of ZEBRA to transiently block p53 function during viral replication and thus alter viral growth properties, accounting in part for the recently described association of ZEBRA with increased mutagenic and carcinogenic properties in a murine model[14]. For example, the limited sequence polymorphism data available for the ZANK region of ZEBRA currently suggests that a mutation in tumor associated ZEBRA from Akata cells is different from canonical B-958 ZEBRA at a single amino acid in the ZANK region (ZEBRA exon 3). Remarkably, primary sequence comparison between ZANK, IκB and related proteins and ASPP2 ankyrins reveals that the substitution of a serine for an alanine at amino acid 205 (Figure 3) is not random but rather converts ZANK from an IκB-like AAA motif to a p53BP2/ASPP2-like ASA motif, also shared with a Cactus ankyrin (Figure 5). Unlike alanine, a serine residue may be covalently modified *in vivo *by phosphorylation, further extending the possible functional consequences of this polymorphism[35]. These observations suggest further study of ZANK polymorphisms *in vivo *and their effects on NF-κB and p53 binding *in vitro *could identify associations between certain EBV strain polymorphisms and cancer phenotypes.

### A model of antagonistic interactions between ZANK NF-κB and p53 protein binding and ZEBRA fos/jun DNA binding

**Figure 13:**
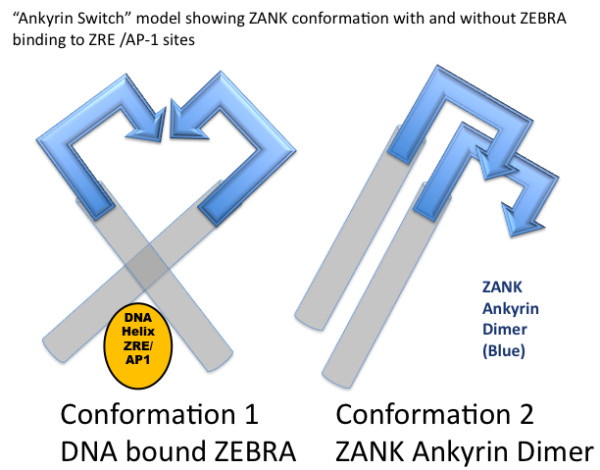
**ZANK Ankyrin Switch Model**. ZEBRA is proposed to have two distinct conformations depending on subcellular localization and DNA-binding. Left: When ZEBRA is bound to ZRE or AP-1 sites on DNA the two alpha helix strands are forced apart similar to the blades of an open scissors, preventing alignment of ZANK domains. Right: When ZEBRA is not bound to DNA either because of its cytoplasmic location or due to its binding to NF-κB and/or p53 proteins, the two alpha helices of the ZEBRA dimer can adopt a parallel or closed conformation similar to the parallel helices of ankyrin bound to NF-κB and/or p53 protein.

As outlined in this manuscript, the extreme carboxyl terminal exon of ZEBRA encodes an amino acid sequence termed ZANK that is functionally and evolutionarily distinct from DNA binding regions of ZEBRA protein. The distinct origin and structure of ZANK suggest that ZANK domain could have a regulatory role on the DNA binding regions of the ZEBRA protein in addition to cytoplasmic effects on NF-κB and p53 proteins (Figure 1). In particular, ZANK could modulate ZEBRA DNA binding through conformational changes in the Fos/Jun like region of ZEBRA (Figure 4) triggered by binding between ZANK and proteins of the NF-κB and p53 family and *vice versa*, modulating the transcriptional activity of the cellular transcription machinery (Figure 13).

Binding of ZANK to proteins of the NF-κB and p53 families could stimulate, inhibit or have no effect of ZEBRA binding to DNA. As shown in Figure 13, structural considerations based on the partial structure of ZEBRA and ZANK support an antagonistic role between ZEBRA DNA binding and ZANK interactions with NF-κB and p53 proteins. Based upon the crystal structure of ZEBRA bound to a ZRE/AP1 DNA binding oligonucleotide (Figure 1), it is apparent that DNA bound to ZEBRA causes the two DNA binding alpha helices to move away from each other. The movement of the ZANK regions at the termini of the helices in a region not bound to DNA is analogous to the blades of a scissor in the open position. A parallel orientation of the ZANK stems, similar to the closed blades of a scissor is required to form an ankyrin-like dimer capable of binding adjacent regions of NF-κB and p53 proteins (Figure 7).

Thus, ZANK could function as a NF-κB and p53 protein dependent switch in the nucleus of EBV infected B-lymphocytes, turning ZEBRA DNA binding off in the presence of high levels of nuclear NF-κB and p53, and conversely activating ZEBRA DNA binding in the absence of nuclear NF-κB and p53 protein (Figure 13). In physiologic terms, these observations are consistent with high levels of active NF-κB and p53 proteins present in the nucleus of latent B-lymphocytes during viral latency, which during viral latency would tend to block ZEBRA DNA binding and prevent activation of viral lytic growth. Conversely, transiently decreased levels of nuclear NF-κB proteins in latently infected B-lymphocytes, due for example to cell stress or starvation could permit increased ZEBRA DNA binding and activation of viral lytic growth, coupling levels of cytoplasmic growth signals mediated by NF-κB and p53 proteins to the viral life cycle.

Later in the viral lytic replication cycle, the NF-κB and p53 protein binding function of ZANK could further trigger cellular apoptosis and facilitate infectious viral release directly through cytoplasmic effects on NF-κB and p53 protein. In T lymphocytes and possibly other non B-lymphocyte cells infected by EBV but incapable of supporting stable viral latency, ZEBRA is almost entirely present in the cell cytoplasm. In these cells, ZEBRA could initially inhibit NF-κB and cause activation of p53 protein and cellular apoptosis as a mechanism of viral immunosuppression.

Transpliced RAZ, containing ZANK but not capable of ZEBRA DNA binding would then serve as an amplifier of the NF-κB and p53 protein functions of ZEBRA, and a corresponding RAZ-like function might explain the non-DNA binding functions of a ZEBRA homologue in HSV-8, the etiologic agent of Kaposi's sarcoma and other human cancers. These possibilities could in principle be tested *in vitro *and *in vivo *through ZEBRA with ZANK domains engineered for either increased or reduced effects on NF-κB and/or p53 protein through mutation of conserved ZANK amino acids interacting with NF-κB and p53 protein identified with the structural models and analysis presented in this work.

## Conclusions

Characterization of B cell lympho-proliferative disorders haa demonstrated that EBV viral latency proteins can transform and immortalize B-lymphocytes *in vitro *and *in vivo*. A causal role of the virus in carcinogenesis is suggested in some tumors because the viral genome sometimes remains in the malignant cell. However, the mechanism of viral carcinogenesis cannot simply be a function of cellular immortalization by the virus since although the vast majority of humans harbor EBV immortalized B lymphocytes, EBV-genome associated cancers are rare [36]. EBV infection may thus also be associated with carcinogenesis not only through cellular immortalization but also through transient mechanisms including destabilization of host genomes[37-40].

A novel role of ZEBRA and ZANK in viral carcinogenesis is suggested in this work. ZEBRA plays a critical role in the viral latent to lytic switch, possibly in part through response to NF-κB and p53 activities by ZANK (Figure 13). Alternating cycles of partial lytic growth triggered by ZEBRA activation and ZEBRA DNA binding followed by ZANK interactions with NF-κB and p53 could synergize with genomic instability contributed by cellular or viral recombinase activation in EBV infected cells. Partial cycles of viral activation and inactivation without completion of the viral life cycle could be particularly relevant in the "hit and run" scenario, proposed for viral carcinogenesis and immune dysregulation as proposed in epithelial breast cancers [41-47].

Notably, it is possible that viral inactivation of p53 originates indirectly or as an evolutionary "spandrel." A spandrel refers to a new phenotype generated by alteration of a related developmental pathway. Because of the evolutionary relationship between NF-κB and p53 through descent from a common ancestral transcription factor, an unavoidable consequence of the inactivation of NF-κB by an ankyrin-like region of ZEBRA is a spandrel-like transient inactivation of p53. Restated, the common origins of NF-κB and p53 conserve a similar ankyrin binding region in both proteins, so any viral protein that targets this site on NF-κB proteins could, in parallel, affect p53 generating new p53 dependent phenotypes. This may in part contribute to the selective loss of the IκBα locus in tumors, a conserved feature of several EBV associated tumors[48,49]. The ZANK spandrel effects on p53 are maintained because of a selective benefit to the virus from related transient inactivation of the NF-κB transcription family and the innate immune system, as well as effects of ZANK on regulation of ZEBRA DNA binding.

Mutant p53 proteins were first characterized as oncogenes because their presence was associated with tumor phenotypes including tumor growth and resistance to apoptosis[50-52]. Surprisingly, wild type p53 was later shown to be a tumor suppressor through the effects of the protein as a DNA-binding transcription factor, rather than a tumor promoter. These contradictory effects could result from opposing tumor promoting spandrel effects between numerous viral proteins such as ZEBRA and mutant p53 proteins, versus tumor suppressing DNA binding gene activation by wild type p53 protein. Thus ZANK might augment the effects of mutant p53, not directly related to p53 DNA binding, but rather due to effects on structurally similar regions of NF-κB proteins required for cellular proliferation.

From an evolutionary perspective, it is likely that EBV encoded ZEBRA protein and a related protein in Human Herpes Virus 8 diverged from other BZIP DNA binding proteins such as Fos/Jun through viral capture of a terminal exon encoding a single ankyrin-like stem and loop. This domain was capable of dimerization into a structure resembling adjacent IκB ankyrins, and was subject to positive selection through interactions with NF-κB proteins. However, because of the shared ankyrin binding domain between NF-κB and p53 proteins an unfortunate consequence of NF-κB inhibition by ZANK would also unavoidably be inactivation of p53. This could be particularly important as a pathogenic mechanism of EBV-related genomic instability in epithelial cells and T lymphocytes that do not support the establishment of viral latency or replication but can sustain a limited form a viral lytic growth that includes high levels of expression of a trans-spliced protein "RAZ" containing ZANK, but without the DNA-binding transcriptional activity on ZRE. In this context, these cells are "dead end hosts" in their inability to support viral replication but can nevertheless suffer the pathophysiological and molecular consequences of viral infection.

Viral pre-conditioning by common viral pathogens has been indirectly implicated in the clustering of human cancers both geographically and temporally based upon indirect evidence in human cancers that defective or abortive replication of common viral pathogens can account for clustering of cancers[53]. In the "viral conditioning" scenario, abortive cycles of viral replication can promote genomic instability. A model is presented here for the specific case of EBV. In the case of EBV and, potentially, other common viruses that interact with both NF-κB and p53, transient lytic gene expression in both B lymphocytes and non-B cells could provide a mechanism for this viral pre-conditioning where defective or aborted viral replication contributes to malignancy as supported by indirect epidemiologic evidence[54-56]. These effects of ZANK could be of particular importance not only in B lymphocytes but in epithelial and T cell infection in which the virus expresses lytic gene products but in most cases does not establish latency[57]. In these cell types transient inactivation of the tumor suppressor p53 would occur in the context of activation of endogenous somatic recombination pathways, potentially resulting in re-arrangement, instability and mutation of endogenous oncogenes.

In summary, we have demonstrated that ZEBRA is a hybrid protein resulting from viral capture of both a Fos/Jun like transcription factor and a polymorphic IκB-like ankyrin carboxyl terminus. Primary sequence and structural similarities are evident between the carboxyl terminus of ZEBRA protein and the conserved stem regions of IκBα and p53BP2/ASPP2 ankyrins. It is suggested that a shared binding pocket in NF-κB is in turn bound by all of these ankyrin proteins and that ZANK is analogous to a "master key" capable of fitting into both NF-κB and p53 protein "locks". In the case of ZEBRA, protein binding to NF-κB provides a selective advantage to the virus through inactivation of the immune response against the virus during viral lytic replication, simultaneously and unavoidably altering the activation of p53.

## Competing interests

The authors declare that they have no competing interests.

## Authors' contributions

DHD designed and executed experiments confirming interactions between ZEBRA, p53, and NF-κB, identified similarities between ZEBRA and ankyrin amino acids, conceived and executed Pymol models and other artwork, drafted manuscript, and paid costs of manuscript processing. YL, JTC conceived and executed statistical analysis and alignments of ZANK and related ankyrins, and edited the manuscript.

LYG provided historical background and terminology of previous research in viral carcinogenesis, p53, and NF-κB, and ankyrin proteins, designed and executed experiments confirming interactions between ZEBRA, p53, and NF-κB, identified similarities between ZEBRA and ankyrin amino acids, and edited the manuscript and artwork. All authors read and approved the final manuscript.
